# Effective Surgical Site Infection Prevention Strategies for Diabetic Patients Undergoing Surgery: A Systematic Review

**DOI:** 10.7759/cureus.59849

**Published:** 2024-05-07

**Authors:** Shenouda Abdallah, Sabri M Hammoud, Hamza Al Balushi, Muhammad M Loon, Yoalkris E Salcedo, Muhammad Mustaneer Ul Hassan, Muhammad J Cheema, Faizan Kadri, Abdullah Shehryar, Abdur Rehman, Muhammad Ibrahim

**Affiliations:** 1 Surgery, Sheikh Jaber Al-Ahmad Al-Sabah Hospital, Kuwait City, KWT; 2 General Surgery, Sheikh Jaber Al-Ahmad Al-Sabah Hospital, Kuwait City, KWT; 3 Medicine, First Bethune Hospital, Muscat, OMN; 4 Surgery, Mayo Hospital, Lahore, PAK; 5 Surgery, Ibero-American University, Santo Domingo, DOM; 6 General Surgery, Sir Ganga Ram Hospital, Lahore, PAK; 7 Medicine, Nantong University, Nantong, CHN; 8 Internal Medicine, Allama Iqbal Medical College, Lahore, PAK; 9 Medicine, Jinnah Hospital, Lahore, PAK

**Keywords:** surgical site infections (ssis), ssi prevention strategies in diabetic surgical patients, diabetes, peer-reviewed clinical trials, prevention strategies, surgical site infections, cochrane library, embase, medline

## Abstract

Surgical site infections (SSIs) pose a significant clinical challenge, with heightened risks and severe consequences for diabetic patients undergoing surgical procedures. This systematic review aims to synthesize the current evidence on effective prevention strategies for mitigating SSI risk in this vulnerable population.

From inception to March 2024, we comprehensively searched multiple electronic databases (PubMed, Medline, Embase, Cochrane Library, CINAHL) to identify relevant studies evaluating SSI prevention strategies in diabetic surgical patients. Our search strategy followed Preferred Reporting Items for Systematic Reviews and Meta-Analysis (PRISMA) guidelines, utilizing a combination of keywords and Medical Subject Headings (MeSH) terms related to diabetes, surgical site infections, prevention strategies, and surgical procedures. Inclusion criteria focused on peer-reviewed clinical trials, randomized controlled trials, and meta-analyses published in English.

The search yielded three studies meeting the eligibility criteria, subject to data extraction and qualitative synthesis. Key findings highlighted the efficacy of interventions such as optimized perioperative glycemic control, timely prophylactic antibiotic administration, and meticulous preoperative skin antisepsis in reducing SSI rates among diabetic surgical patients. The potential for personalized prevention approaches based on individual patient factors, such as diabetes type and surgical complexity, was explored.

This systematic review underscores the importance of a multifaceted, evidence-based approach to SSI prevention in diabetic surgical patients, integrating strategies like glycemic control, antibiotic prophylaxis, and preoperative skin antisepsis. Furthermore, our findings suggest the potential benefits of personalized care pathways tailored to individual patient characteristics. Implementing these interventions requires interdisciplinary collaboration, adaptation to diverse healthcare settings, and patient engagement through culturally sensitive education initiatives. This comprehensive analysis informs clinical practice, fosters patient safety, and contributes to the global efforts to enhance surgical outcomes for this high-risk population.

## Introduction and background

Surgical site infections (SSIs) pose a significant threat to patient safety and healthcare quality, with profound implications for postoperative morbidity, mortality, and escalating healthcare costs [1]. This formidable challenge is further exacerbated in patients with diabetes mellitus, a prevalent metabolic disorder characterized by impaired glucose regulation and heightened susceptibility to infections [2]. Diabetes has been identified as a significant risk factor for SSIs, with mounting evidence suggesting a multifactorial interplay between hyperglycemia, impaired wound healing, and compromised immune function, contributing to increased infection rates [3].

The global burden of SSIs among diabetic surgical patients is staggering. A large-scale meta-analysis by Martin et al. (2015) [3] revealed that diabetic patients undergoing various surgical procedures have a 53% higher risk of developing SSIs compared to their non-diabetic counterparts. This risk amplifies in specific surgical contexts, such as cardiac surgeries, where diabetic patients exhibit a two-fold increased risk of SSIs [3]. Furthermore, the economic toll of SSIs in this patient population is substantial, with estimates suggesting that diabetic patients with SSIs incur significantly higher healthcare costs and longer hospital stays compared to those without infections [4].

Recognizing the gravity of this issue, numerous strategies have been proposed and implemented to mitigate SSI risk in diabetic surgical patients. These interventions span various approaches, including optimal perioperative glycemic control, prophylactic antibiotic administration, meticulous preoperative skin antisepsis, and emerging technologies such as antimicrobial dressings and negative pressure wound therapy [5]. However, these strategies' relative efficacy and practicality remain subjects of ongoing debate, with varying degrees of success reported across different healthcare settings and patient demographics.

In light of the significant clinical and economic burden posed by SSIs in diabetic surgical patients, this systematic review aims to critically synthesize and evaluate the current evidence on effective prevention strategies for this high-risk population. By rigorously appraising the existing literature, our overarching objective is to comprehensively analyze the most promising interventions, their potential for personalization based on individual patient factors, and the practical considerations for implementing these strategies across diverse healthcare settings worldwide. Ultimately, this review seeks to inform evidence-based clinical practice, foster interdisciplinary collaboration, and contribute to global efforts to enhance surgical outcomes and improve the quality of care for diabetic patients undergoing surgical interventions.

## Review

Materials and methods

Search Strategy

Our search strategy was meticulously developed in adherence to the Preferred Reporting Items for Systematic Reviews and Meta-Analysis (PRISMA) guidelines to identify studies evaluating effective surgical site infection (SSI) prevention strategies for diabetic patients undergoing surgery. To ensure a comprehensive retrieval of relevant literature, extensive searches were conducted across several key electronic databases: PubMed, Medline, Embase, the Cochrane Library, and CINAHL. The time frame for our search extended from the inception of each database to March 2024.

We constructed a combination of keywords and Medical Subject Headings (MeSH) terms related to our research question, including "diabetes mellitus", "surgical site infection", "SSI prevention", "surgery", and "randomized controlled trials". Boolean operators ('AND', 'OR') combined these terms and enhanced the search effectively. Example search strings included: "diabetes mellitus AND surgical site infection AND prevention", "diabetic patients AND surgery AND SSI prophylaxis", and "antibiotic prophylaxis OR infection control AND diabetic surgical patients".

To broaden our search and capture the maximum number of studies, we also examined the reference lists of all selected articles for additional pertinent studies. Furthermore, our search strategy extended to include clinical trial registries and relevant conference proceedings to identify unpublished or ongoing research in this area. To ensure the robustness and comprehensiveness of our search strategy, it was reviewed by an expert in medical information retrieval, with experience specific to infection prevention in surgical settings.

We limited our search to studies published in English and peer-reviewed. Our inclusion criteria were specifically designed to identify clinical trials, meta-analyses, and randomized controlled trials that focused on the efficacy of various SSI prevention interventions in diabetic patients undergoing surgery.

Eligibility Criteria

The eligibility criteria for this systematic review have been meticulously defined to ensure the rigor and relevance of the included studies. Our review targets peer-reviewed research articles, including clinical trials, randomized controlled trials (RCTs), and meta-analyses, that concentrate on surgical site infection (SSI) prevention strategies in diabetic patients undergoing surgical procedures.

Inclusion criteria for the selection of studies in this review encompass a range of parameters to ensure the relevance and reliability of the gathered evidence. Only peer-reviewed research articles, spanning cohort studies, clinical trials, and meta-analyses, are considered eligible for inclusion. The focus is specifically on diabetic patients undergoing surgical procedures, emphasizing interventions directed toward preventing surgical site infections (SSIs). These interventions encompass a broad spectrum, including but not limited to antibiotic prophylaxis, glycemic control measures, and preoperative skin preparation techniques. To maintain consistency and facilitate thorough analysis, studies published in English from the databases' inception until April 2024 are included to incorporate the most contemporary findings into the review.

Conversely, exclusion criteria serve to delineate boundaries and filter out studies that do not align with the primary objectives of the review. Studies not directly investigating SSI prevention strategies in diabetic surgical patients are excluded to maintain relevance. Similarly, research solely based on animal models is omitted to ensure a focus on outcomes directly applicable to human patients. Grey literature, such as conference abstracts and unpublished works, is excluded from upholding standards of rigor and reliability, prioritizing peer-reviewed publications. Non-English language studies are also excluded due to potential challenges in translation and interpretation, which could compromise the accuracy of findings. Finally, studies lacking sufficient detail on intervention methods or outcomes related to SSI prevention in diabetic surgical patients are excluded to maintain the integrity and depth of the analysis.

Data Extraction

Our data extraction process was meticulously designed to ensure the reliability and validity of the data collected for our systematic review on effective SSI prevention strategies for diabetic patients undergoing surgery. Initially, we screened articles based on their titles and abstracts. Two independent reviewers evaluated these for relevance, classifying them as "relevant", "not relevant", or "probably relevant". This preliminary assessment was crucial for honing in on the most pertinent articles for our review focus.

Following this, articles deemed potentially eligible from the initial screening underwent a full-text review. To ensure consistency across the data collection process, we conducted the data extraction using a standardized form in Microsoft Excel (Microsoft, Redmond, Washington). Each reviewer independently utilized this form, applying the predefined inclusion and exclusion criteria to each article. In discrepancies or disagreements between the reviewers, a third reviewer intervened to resolve these issues through discussion, thereby maintaining accuracy and consistency in our selection process.

The data extraction form was designed to capture essential information critical to our review's objectives, including the lead author's name, publication year, study design, population size, key findings, and noted limitations. This structured approach enabled a comprehensive analysis of each study, ensuring all relevant information was meticulously considered and synthesized for our review.

Data Analysis and Synthesis

Given the heterogeneity of the studies involved, we decided not to pursue a meta-analysis. Instead, our review's data analysis and synthesis were tailored to qualitatively assess and integrate the findings from the selected studies. This approach allowed for a detailed exploration of the effectiveness of various SSI prevention strategies in diabetic patients undergoing surgery, drawing from the specific contexts and outcomes reported in each study.

We categorized the key findings from each study to identify common themes and notable differences regarding SSI risks, prevention strategies, and outcomes in diabetic surgical patients. This thematic synthesis provided insights into the factors contributing to increased SSI risks in this population and highlighted the prevention strategies showing the most promise.

Our narrative synthesis integrated these findings, offering a holistic view of the current state of research on SSI prevention in diabetic patients undergoing surgery. We discussed the implications of these findings in the broader context of surgical care for diabetic patients, identified gaps in the current literature, and suggested directions for future research.

This qualitative synthesis not only highlighted the correlations and divergences among the collected data but also assessed the quality and strength of the evidence. By doing so, we contributed valuable insights into the effective prevention of SSIs in diabetic surgical patients, underscoring the need for targeted strategies to mitigate these risks.

Results

Study Selection Process

**Figure 1:**
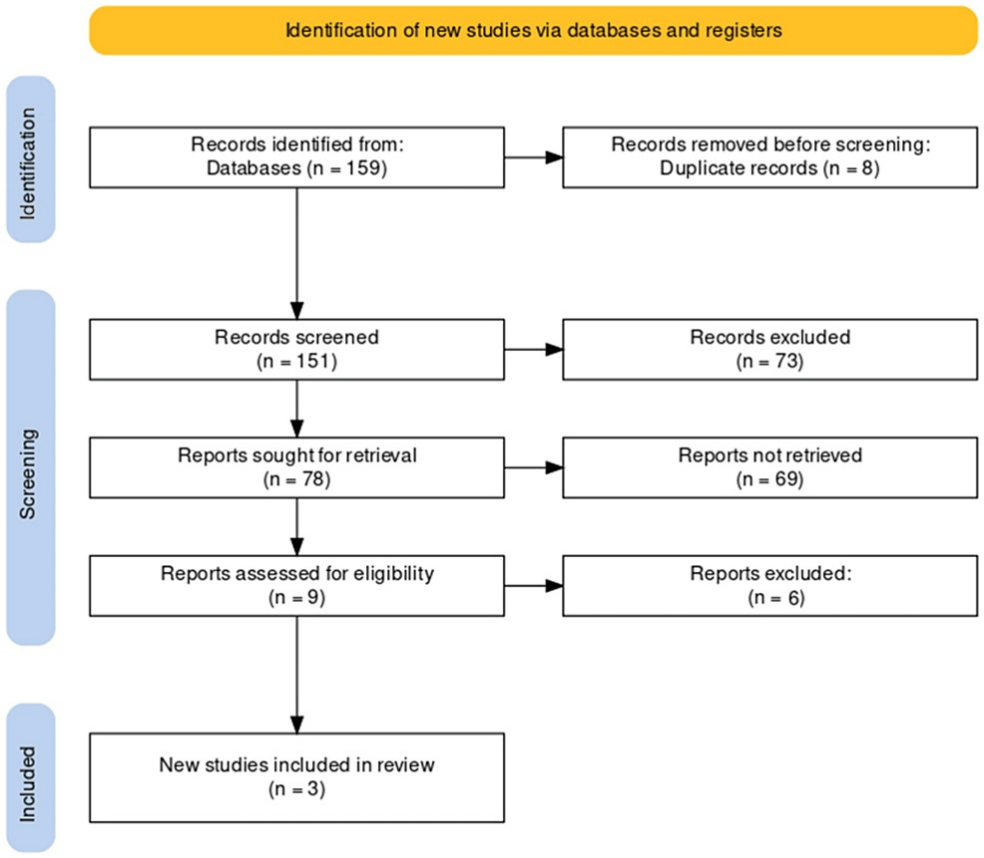
PRISMA flow diagram of the selection of studies for inclusion in the systematic review PRISMA - Preferred Reporting Items for Systematic Reviews and Meta-Analysis (PRISMA)

The search across multiple databases resulted in the identification of 159 records. After removing eight duplicates, 151 records remained. These were screened, leading to the retrieval of 78 reports for further assessment. Upon closer examination for eligibility, nine reports were considered, and three new studies met the inclusion criteria and were selected for inclusion in the systematic review. The PRISMA flowchart provided in Figure 1 visualizes this study selection process clearly and methodically.

Characteristics of Selected Studies

Our systematic review evaluated three seminal studies, each offering substantial evidence on the impact of diabetes on surgical site infection (SSI) risk. The first, by Martin et al. in 2015, was a meta-analysis of 94 articles that determined a notable association between diabetes and higher SSI risk, particularly in cardiac surgeries. Shiferaw et al.'s 2020 study synthesized data from 24 studies to report a 12.3% pooled prevalence of SSIs in Ethiopia, identifying surgery duration and diabetes as significant risk factors. Wu et al., in 2021, examined 19 studies, highlighting the increased complications in diabetic patients undergoing total joint arthroplasty. While these studies are groundbreaking, they acknowledge limitations such as selection bias, variability in SSI definitions, and heterogeneity in study designs. The summary is provided in Table 1.

**Table 1:** The table summarizing key studies included in the review. SSI - surgical site infection; OR - odds ratio; BMI - body mass index; ASA score - American Society of Anesthesiologists score; Preop - preoperative; TJA - total joint arthroplasty; IDD - insulin-dependent diabetes; IDDM - insulin-dependent diabetes mellitus; NIDDM - non-insulin-dependent diabetes mellitus

Author	Year	Study design	Population size	Key findings	Limitations
Martin et al. [3]	2015	Systematic Review and Meta-analysis	94 articles met inclusion criteria after reviewing 3,631 abstracts and 522 full texts	Diabetes associated with higher SSI risk (OR=1.53) Stronger association for cardiac surgery (OR=2.03) SSI class, study design, patient BMI did not impact results	Include study selection bias, variability in SSI definitions, reporting across studies
Shiferaw et al. [6]	2020	Systematic Review and Meta-analysis	24 studies, 13,136 participants	Pooled SSI prevalence in Ethiopia 12.3% Significant risk factors: surgery >1hr, diabetes, ASA score >1, previous surgery, contaminated wound, preop stay >7 days	Publication bias, heterogeneity, limitations of included studies
Wu et al. [7]	2021	Systematic Review and Meta-(Regression) Analysis	19 studies, 85,689 participants	26% of diabetic TJA patients had IDD IDDM increased risks of complications like cardiac arrest, renal failure, deep SSI, dehiscence, death vs NIDDM Increasing IDDM prevalence time trend	Include variability in study designs/measures, publication bias, limitations in diabetes management data

Discussion

Our systematic review meticulously synthesizes findings from a select yet impactful collection of studies, shedding light on the multifaceted dynamics of surgical site infection (SSI) prevention strategies in diabetic patients undergoing surgery. The cornerstone of our review rests upon the comprehensive analyses presented by Martin et al. [3], Shiferaw et al. [6], and Wu et al. [7], which collectively provide a panoramic view of the current state of research in this domain.

The review by Martin et al. revealed a significant association between diabetes and increased risk of SSI, particularly highlighting a heightened risk in cardiac surgery patients. This pivotal finding underscores the critical need for tailored SSI prevention strategies in diabetic populations. Shiferaw et al. extended the discourse by quantifying the pooled SSI prevalence in Ethiopia, revealing an alarming rate of 12.3%, alongside identifying key risk factors such as extended surgery duration and preoperative hospital stays exceeding seven days. These insights highlight the global burden of SSIs and pinpoint specific modifiable risk factors that could be targeted in prevention protocols. Lastly, the work by Wu et al. delves into the specific context of total joint arthroplasty (TJA), demonstrating the exacerbated risks faced by insulin-dependent diabetic patients, thereby advocating for nuanced management strategies to mitigate these risks.

Collectively, these studies illuminate the intricate interplay between diabetes and SSI risk, emphasizing the need for proactive and diabetes-specific SSI prevention strategies. While each study contributes unique insights into various aspects of SSI prevention, they underscore a universal imperative: developing and implementing robust, evidence-based, and contextually relevant SSI prevention protocols to safeguard diabetic surgical patients against the multifarious risks of postoperative infections.

Our systematic review explains the complexities of surgical site infection (SSI) prevention in diabetic patients, affirming and expanding upon previous studies while identifying divergent findings for future exploration. Echoing Hweidi et al. [8], our analysis emphasizes the importance of glycemic control and identifies critical interventions like perioperative antibiotic prophylaxis and preoperative skin antiseptics. It corroborates de Lissovoy et al. [9] regarding the risk increase associated with longer preoperative hospital stays, with our data adding depth through various geographical contexts. Contrasts emerged around insulin dependency's impact, where our findings suggest a nuanced risk differential between insulin-dependent and non-insulin-dependent diabetes, challenging the generalizations made by Wu et al. [7] and prompting a reassessment of diabetes's role in SSI risk across different surgeries.

Further, our review enriches the dialogue on SSI risk factors, providing a detailed examination of surgery duration, wound classification, and postoperative glycemic control's interrelations, as initially touched upon by Martin et al. [3] and Shiferaw et al. [6]. By doing so, it underscores the multifaceted nature of SSI risks in diabetics and highlights research gaps, particularly in assessing new SSI prevention technologies. Our findings advocate for a personalized, evidence-driven approach in clinical practice, emphasizing the need for ongoing, nuanced research to enhance SSI prevention strategies for diabetics undergoing surgery, thus pointing towards areas requiring further investigation to refine effective prevention measures.

This systematic review delineates the essential components and clinical implications of preventing surgical site infections (SSIs) in diabetic patients, underscoring the multifaceted, evidence-based strategies required for effective management. It highlights the pivotal role of optimizing perioperative glycemic control, as evidenced by studies [10,11], advocating for a collaborative approach involving surgical teams, endocrinologists, and diabetes educators to tailor individualized glycemic management plans. The significance of prophylactic antibiotic use and rigorous preoperative skin antisepsis is emphasized [5,12], pointing to their effectiveness in reducing SSI rates, especially in high-risk diabetic populations.

Further, the review identifies the importance of personalizing SSI prevention strategies based on patient-specific factors, such as diabetes type and surgical procedure [13,14]. This approach is particularly crucial for insulin-dependent diabetics undergoing complex surgeries, suggesting a need for stricter protocols and monitoring. The adaptation of these strategies in diverse healthcare environments calls for a comprehensive plan that addresses resource availability, healthcare policies, and patient demographics. Innovations in antibiotic stewardship, cost-effective glycemic monitoring, and infection control education, especially in resource-constrained settings, are critical [15].

Moreover, the review stresses the significance of culturally sensitive patient education and engagement [16], which is vital for enhancing adherence to SSI prevention measures. Empowering patients with knowledge of glycemic control, wound care, and prophylactic regimens is key to improving prevention outcomes. Thus, the findings advocate for a concerted, interdisciplinary effort, accommodating the nuances of various healthcare settings and patient backgrounds, to implement and sustain effective SSI prevention strategies in diabetic surgical patients, emphasizing the need for ongoing innovation and collaboration in this field.

This systematic review highlights actionable insights for clinicians and healthcare professionals on preventing surgical site infections (SSIs) in diabetic patients, emphasizing the importance of standardized perioperative glycemic control protocols. It advocates for healthcare institutions to prioritize developing and disseminating evidence-based guidelines tailored to individual patient factors, such as diabetes type, comorbidities, and surgical procedures [11]. Additionally, the necessity of strict adherence to prophylactic antibiotic administration and preoperative skin antisepsis protocols is underscored, urging the establishment of clear guidelines and monitoring systems for compliance with best practices [17].

Moreover, the review suggests the benefits of personalized SSI prevention approaches, encouraging healthcare professionals to tailor strategies to patient-specific risk factors, including insulin dependence and the complexity of the surgical procedure [18]. Effective implementation of these recommendations requires interdisciplinary collaboration among surgeons, endocrinologists, infection control specialists, and nursing staff. By fostering multidisciplinary teams and communication, healthcare providers can develop and apply comprehensive SSI prevention protocols, enhancing patient outcomes and advancing the fight against SSIs in diabetic surgical patients.

Our systematic review has revealed critical gaps in current research on surgical site infection (SSI) prevention in diabetic patients, underscoring the urgent need for broader, more detailed studies. Specifically, there is a conspicuous absence of large-scale, multicentric randomized controlled trials (RCTs) that assess the efficacy of various SSI prevention strategies across diverse populations and healthcare settings. This limitation restricts the applicability and generalizability of existing findings, highlighting the necessity for future research to encompass well-designed RCTs that span different patient demographics, surgical procedures, and healthcare systems [19,20]. Additionally, the long-term effectiveness of these interventions remains underexplored, with a dearth of longitudinal studies examining their sustainability and impact over time on patient health, healthcare costs, and quality of life [21,22].

Moreover, implementing SSI prevention strategies in resource-constrained settings poses significant challenges, necessitating research on cost-effective, adaptable methods suitable for such environments. Identifying barriers to implementing evidence-based strategies in low-resource settings and investigating innovative, context-specific solutions are crucial steps forward [23]. There's also a call for research into personalized prevention approaches, considering the intricate relationship between diabetes specifics, surgical factors, and SSI risk, to develop tailored, precision-based protocols. Addressing these gaps through comprehensive, collaborative research efforts is paramount for enhancing SSI prevention in diabetic surgical patients, promising improved healthcare outcomes and a higher standard of patient care globally.

This systematic review serves as a resounding call to action for the global healthcare community to prioritize evidence-based, comprehensive surgical site infection (SSI) prevention strategies tailored specifically for diabetic patients undergoing surgery. Our findings underscore the profound impact that targeted interventions, such as optimized perioperative glycemic control, prophylactic antibiotic administration, and meticulous preoperative skin antisepsis, can have on mitigating the elevated SSI risks this vulnerable patient population faces. By translating these insights into clinical practice through interdisciplinary collaboration, personalized care pathways, and adaptive implementation models, healthcare providers worldwide can substantially improve patient outcomes, reduce healthcare costs, and ultimately enhance the quality of life for countless individuals navigating the intricate intersection of diabetes and surgical care. However, it is crucial to acknowledge the limitations of our review, including the exclusion of non-English language studies and the potential for biases in study selection. The ethical and policy implications of implementing these prevention strategies must be carefully considered, ensuring equitable access and culturally sensitive approaches across diverse healthcare systems and regions. Nonetheless, the educational value and potential global impact of our findings cannot be overstated, as they pave the way for a future where diabetic patients can undergo surgical procedures with the utmost confidence in their safety and well-being.

## Conclusions

This systematic review provides a comprehensive synthesis of the current evidence on preventing surgical site infections (SSIs) among the high-risk population of diabetic surgical patients. By meticulously evaluating and integrating findings from influential studies, our analysis illuminates the importance of a multifaceted, evidence-based approach to SSI prevention in this vulnerable cohort. Strategies such as optimized perioperative glycemic control, timely prophylactic antibiotic administration, and meticulous preoperative skin antisepsis have become crucial components of a comprehensive prevention protocol. Furthermore, our findings underscore the potential benefits of personalized care pathways tailored to individual patient factors like diabetes type and surgical complexity. The successful implementation of these interventions, facilitated by interdisciplinary collaboration and adaptation to diverse healthcare settings, holds immense promise for improving surgical outcomes, reducing healthcare costs, and ultimately enhancing the quality of life for diabetic patients worldwide. This review serves as a clarion call for the global healthcare community to prioritize tailored SSI prevention strategies, paving the way for a future where diabetic individuals can navigate the surgical journey with confidence and optimal protection against postoperative infections.
